# Relationship between vitamin D receptor gene polymorphisms (*Bsm*I, *Taq*I, *Apa*I, and *Fok*I) and calcium intake on bone mass in young Japanese women

**DOI:** 10.1186/s12905-021-01222-7

**Published:** 2021-02-19

**Authors:** Yuri Sakamoto, Fumi Oono, Kaoruko Iida, Pao-Li Wang, Yoichi Tachi

**Affiliations:** 1 Department of Clinical Dietetics and Human Nutrition, Faculty of Pharmacy and Pharmaceutical Sciences, Josai University, Saitama, Japan; 2 Department of Nutrition and Food Science, Graduate School of Humanities and Sciences, Ochanomizu University, Tokyo, Japan; 3 Institute for Human Life Innovation, Ochanomizu University, Tokyo, Japan; 4 Department of Innovation in Dental Education, Osaka Dental University, Osaka, Japan; 5 Laboratory of Nutrition Physiology, Tokyo Kasei University, Tokyo, Japan

**Keywords:** Bone mass, Calcium intake, Genotyping, Osteoporosis, Premenopausal women, Vitamin D receptor gene polymorphisms

## Abstract

**Background:**

The high prevalence of low bone mass in young women in Japan has emerged as a serious health issue in recent years. Therefore, the aim of the present study was to reevaluate the relationship between genetic and dietary factors, as well as its influence on bone mass in young Japanese women, with particular emphasis on vitamin D receptor (VDR) gene polymorphisms and calcium intake.

**Methods:**

A total of 499 Japanese women aged 20–24 years were enrolled in the study. The bone mass of the calcaneus was assessed using the quantitative ultrasound method and expressed as the osteo sono-assessment index (OSI). VDR gene polymorphisms (*Bsm*I, *Taq*I, *Apa*I, and *Fok*I) were analyzed using DNA extracted from saliva. Calcium intake was assessed using the Food Frequency Questionnaire based on food groups (FFQg) and adjusted with the energy intake. Participants were divided into two groups based on the median calcium intake (250 mg/1000 kcal).

**Results:**

Consequently, bone mass was significantly different among the *Bsm*I and *Taq*I genotypes after adjusting for body mass index (BMI) (*p* = 0.030 and 0.019, respectively). In addition, the *Bsm*I AA and *Apa*I GT genotypes showed significant differences in bone mass between the calcium-intake groups, with low OSI in the low-calcium intake group and high OSI in the high-calcium intake group, respectively, even after adjusting for BMI (*p* = 0.020 and 0.038, respectively).

**Conclusions:**

These findings may prove instrumental in developing a logical approach towards preventing bone loss in young Japanese women.

## Background

In Japan, as well as in several developed countries, an increasing prevalence of low body weight among the younger generation poses a critical health issue. The body mass index (BMI) of Japanese men and women is the lowest among the BMI values calculated for people of developed countries [1]. Furthermore, according to a study of high school students in Japan, the United States, China, and Korea conducted by the National Institution for Youth Education in 2018, the lowest BMI was computed for Japanese high school students among the youth of all four countries [2]. Approximately 20% of Japanese women in their 20 s are underweight (BMI < 18.5), a rate that has been largely static over the past decade [3].

In women, menopause results in the rapid decline of bone mass (or density) owing to reduced estrogen production [4, 5]. Furthermore, BMI demonstrates a strong positive correlation with bone mineral density (BMD) [6–8]. It is known that excessive weight loss at a young age causes a deterioration in bone mass and accelerates postmenopausal osteoporosis. In a previous study, 17% of the 161 young Japanese women participants (age 19.8 ± 1.1 years) showed low BMD as compared to that of women aged 50–60 years [9]. To impede the progression of osteoporosis, it is crucial to prevent postmenopausal bone loss and also to boost bone mass at a younger age [10]. Attaining a higher bone mass by the end of the growth period can potentially prevent fractures and conserve bone mass [10]. Orito et al. [11] reported that the BMD of young Japanese women peaked at 18–29 years. Thus, augmenting BMD in women in this age range is important for maintaining bone health and preventing osteoporosis throughout their lifetime.

Bone mass is influenced by genetic factors, which determine approximately 80% of bone mass [12]. Previous studies on the bone density of twins reported that monozygotic twins demonstrated a greater degree of similarity in bone density as compared to dizygotic twins [13–15]. One such genetic factor is single-nucleotide polymorphism (SNP). Vitamin D and its receptors are involved in calcium metabolism, and bone density is reportedly influenced by the associated genotypes, e.g., *Bsm*I (rs1544410), an SNP in the vitamin D receptor (VDR) genes [14]. In their meta-analysis, Gong et al. [16] reported that VDR genotypes and bone density were related; the obtained results differed depending on factors such as gender, age, postmenopausal age, and the presence/absence of osteoporosis. In addition, lifestyle-related factors, including dietary calcium intake, exercise, smoking, and alcohol consumption, can also affect bone mass during growth [12, 17, 18]. Thus, it is important to understand the relationship between bone mass and the VDR gene polymorphisms in the participants, relative to other factors.

Several prior studies, also conducted in Japan, have investigated the effect of genetic and environmental factors on bone loss related to menopause. In contrast, there are relatively few studies that assessed the relationship among bone mass, VDR gene polymorphisms, and environmental factors in premenopausal Japanese women. Moreover, these studies typically contained a low number of participants with a relatively wide age range (18–44 years) [19–21]. Estrogen levels and lifestyle factors among participants can vary if a wide age range is considered in a study. Therefore, the present study aimed to investigate how VDR gene polymorphisms and calcium intake affect bone mass in a narrow-age cohort of Japanese women in their early twenties.

## Methods

### Study participants

**Fig. 1:**
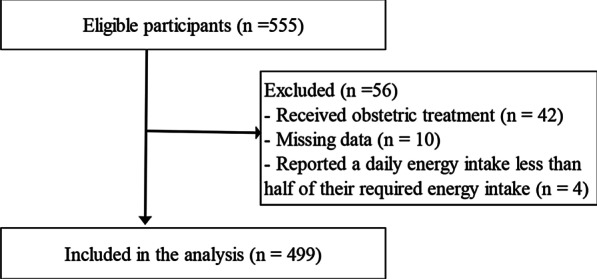
Flow diagram of participant inclusion and exclusion

Eligible participants comprised women aged 20–24 years enrolled at Tokyo Kasei University. The study was conducted between April 2015 and March 2019, targeting third-year university students and second-year junior college students in every October, second-year junior college students in every March. Sample size calculation was not performed in this study. Written and verbal consent was obtained from 555 women who were given detailed explanations regarding bone mass measurements, questionnaires, and saliva collection. Participants who received obstetric treatment (n = 42), with missing data (n = 10), or reported a daily energy intake less than half of their required energy intake (n = 4) were excluded. Thus, the final analysis involved the processing and further evaluation of the data obtained from 499 participants (Fig. 1). The study was approved by the ethics committee of Tokyo Kasei University (Approval No. ITAH26-04).

### Calcaneus bone mass measurement

Bone mass at the right calcaneus was indirectly measured by quantitative ultrasound (QUS) using an AOS-100SA system (Hitachi Ltd., Tokyo, Japan); it is an indirect measure of bone mass. The correlation between bone mass (measured using QUS) and bone mineral density (measured by dual-energy X-ray absorptiometry) was strong (r = 0.804, *p* < 0.001) [22]. Furthermore, QUS is a non-invasive, painless method that does not involve exposure to radiation [22]. It is commonly carried out in Japan to screen patients for osteoporosis. Speed of sound (SOS) and transmission index (TI) were measured, and the osteo sono-assessment index (OSI) was calculated as the following formula: OSI = TI × SOS^2^. OSI was used as an indicator of bone mass. All OSI values were divided by 10^6^ and used in the study.

### DNA extraction and genotyping

Saliva samples were collected from each participant using Oragene^®^ DNA OG-500 (DNA Genotek Inc., Ottawa, ON, Canada) and stored at 20–25 °C until further processing for DNA extraction. Genomic DNA was isolated from saliva in accordance with the manufacturer’s protocol. The extracted DNA was diluted with sterile water to a concentration lower than 100 ng/µL and stored at − 20 °C. Genotyping was performed by real-time PCR using the Thermal Cycler Dice^®^ Real Time System (Takara Bio Inc., Shiga, Japan). Genotyping of VDR gene polymorphisms *Bsm*I (rs1544410) and *Taq*I (rs731236) was detected using the Cycleave PCR Reaction Mix (Takara Bio Inc., Shiga, Japan). The genotyping of *Apa*I (rs7975232) and *Fok*I (rs2228570) was conducted using the TaqMan^®^ GTXpress™ Master Mix (Applied Biosystems Inc., Foster City, CA, USA).

### Measurement of dietary calcium intake and BMI

Daily energy and calcium intake were assessed using the commercially available questionnaire, Food Frequency Questionnaire based on food groups (FFQg) Ver.3.0 (Kenpakusha Co. Ltd., Tokyo, Japan), and analyzed using Excel add-in software (Excel Eiyou-kun, Kenpakusha Co. Ltd., Tokyo, Japan). FFQg included questions about 29 food groups (e.g., milk, other dairies, fish and shellfish, small fish, and green and yellow vegetables) and 10 types of cooking. Nutrient intake (including calcium intake) was calculated follows: nutrient intake (unit/day) = specific portion size of each food group × reported categories of portion size × eating frequency (per week)/7 × amount of each nutrient (unit/100 g) of food group/100. The reported categories of portion size were obtained from the question about the categories of portion size of each food group as the following answers: never (0), small (0.5), normal (1), large (1.5). For some food groups which did not include the question on the categories of portion size of each food group, the reported categories of portion size were excluded from the formula. This questionnaire has been widely used for the research in Japan [23, 24]. Daily energy and calcium intake calculated based on FFQg correlated significantly with that calculated using 7-day weighed dietary records; the evaluation was performed on 66 participants aged 19–60 years (r = 0.465, 0.410, respectively) [25, 26]. Calcium intake was energy-adjusted using the density method to reduce the influence of misreporting [27, 28]. The participants were divided into two groups based on the median energy-adjusted calcium intake. In addition, BMI (kg/m^2^) was calculated based on self-reported height and weight data from FFQg.

### Statistical analysis

All data were statistically evaluated using SPSS version 24 software (SPSS Inc., Chicago, IL, USA). The χ^2^ test was used to compare the frequency of the observed genotypes with the Hardy–Weinberg equilibrium. The characteristics of the genotype groups were comparatively analyzed using analysis of variance (ANOVA). Analysis of covariance (ANCOVA) was used to evaluate the effect of calcium intakes on OSI based on VDR genotypes. The participants were divided into two groups based on high and low calcium intake. The association between VDR genotypes and OSI was evaluated in each group by ANOVA and ANCOVA. A value of *P*-value (*p*) < 0.05 was regarded as statistically significant.

## Results

### Characteristics of the participants

The OSI, an indicator of bone mass, was 2.72 ± 0.26 [means ± standard deviation (SD)] (Table 1). The minimum and maximum OSI values were 2.22 and 3.77, respectively. The OSI measured by the Japan Osteoporosis Society, using the same device, was equivalent in women of the same age group. The genotype frequencies of the four VDR gene polymorphisms (*Bsm*I, *Taq*I, *Apa*I, and *Fok*I) were similar to those reportedly previously in Japanese participants [29–31]. No significant difference was observed in Hardy–Weinberg equilibrium (*p* = 0.606, 0.907, 0.948, and 0.382, respectively). A significant difference in OSI was observed among the *Bsm*I and *Taq*I genotypes after adjusting for BMI (*p* = 0.030 and 0.019, respectively) (Table 2) while no significant difference was observed between the other two genotypes (*Apa*I and *Fok*I) (Table 2).

**Table 1:** Characteristics of the participants (n = 499)

Variable	Value
Age (years)	20.0 ± 0.3
Height (cm)	158.7 ± 5.3
Weight (kg)	51.4 ± 6.0
BMI (kg/m^2^)	20.4 ± 2.0
OSI	2.72 ± 0.26
Energy (kcal/day)	1724 ± 388
Calcium (mg/day)	457 ± 170
Calcium (mg/1000 kcal)	263 ± 69

Values are means ± standard deviation (SD) or percentage

**Table 2:** Genotype frequencies and characteristics according to VDR genotypes (*Bsm*I, *Taq*I, *Apa*I and *Fok*I)

	*Bsm*I (rs1544410)			
	GG	GA	AA	*p*
Number (%)	399 (80.0)	92 (18.4)	8 (1.6)	–
Age (years)	20.1 ± 0.0	20.0 ± 0.0	20.0 ± 0.1	0.901
BMI (kg/m^2^)	20.5 ± 0.1	20.1 ± 0.2	19.4 ± 0.6	0.100
Calcium (mg/1000 kcal)	264 ± 3	262 ± 7	247 ± 11	0.780
OSI Not adjusted	2.73 ± 0.01	2.69 ± 0.02	2.45 ± 0.07	0.009^b^
OSI Adjusted for BMI	2.72 ± 0.01	2.70 ± 0.03	2.49 ± 0.09	0.030^a^

	*Taq*I (rs731236)			
	TT	TC	CC	*p*
Number (%)	389 (78.0)	102 (20.4)	8 (1.6)	–
Age (years)	20.1 ± 0.0	20.0 ± 0.0	20.0 ± 0.0	0.545
BMI (kg/m^2^)	20.4 ± 0.1	20.5 ± 0.2	21.0 ± 0.9	0.622
Calcium (mg/1000 kcal)	266 ± 7	254 ± 12	255 ± 3	0.291
OSI Not adjusted	2.73 ± 0.01	2.69 ± 0.03	2.52 ± 0.07	0.058
OSI Adjusted for BMI	2.73 ± 0.01	2.69 ± 0.02	2.49 ± 0.09	0.019^a^

	*Apa*I (rs7975232)			
	GG	GT	TT	*p*
Number (%)	226 (45.3)	222 (44.5)	51 (10.2)	–
Age (years)	20.1 ± 0.0	20.0 ± 0.0	20.0 ± 0.0	0.357
BMI (kg/m^2^)	20.3 ± 0.1	20.6 ± 0.1	20.4 ± 0.3	0.382
Calcium (mg/1000 kcal)	266 ± 4	265 ± 5	246 ± 6	0.156
OSI Not adjusted	2.71 ± 0.02	2.73 ± 0.02	2.68 ± 0.03	0.458
OSI Adjusted for BMI	2.72 ± 0.02	2.72 ± 0.02	2.68 ± 0.03	0.518

	*Fok*I (rs2228570)			
	CC	CT	TT	*p*
Number (%)	195 (39.1)	222 (44.5)	82 (16.4)	–
Age (years)	20.0 ± 0.0	20.1 ± 0.0	20.0 ± 0.0	0.275
BMI (kg/m^2^)	20.6 ± 0.2	20.2 ± 0.1	20.4 ± 0.2	0.140
Calcium (mg/1000 kcal)	256 ± 5	271 ± 5	259 ± 7	0.084
OSI Not adjusted	2.70 ± 0.02	2.72 ± 0.02	2.74 ± 0.03	0.371
OSI Adjusted for BMI	2.69 ± 0.02	2.73 ± 0.02	2.74 ± 0.03	0.133

Values are means ± standard error (SE) or percentage. The *p* values indicate the significance of difference based on genotype. Continuous variables were assessed by ANOVA

^a^*p* < 0.05

^b^*p* < 0.01

### Calcium intake

Median calcium intake (250 mg/1000 kcal) was used to divide the participants into two groups (low-intake group, ≤ 250 mg/1000 kcal, and high-intake group, ≥ 251 mg/1000 kcal). No significant difference was observed in participant characteristics, including the OSI, between the groups (OSI, low-intake group, 2.70 ± 0.27, and high-intake group, 2.73 ± 0.25; *p* = 0.208).

### VDR genotypes, calcium intake, and OSI

OSI was analyzed between VDR genotypes and the calcium-intake groups. A significant difference in the OSI of the calcium-intake groups was observed for the *Bsm*I AA genotype, even after adjusting for BMI (*p* = 0.011 and 0.020, respectively) (Table 3). A significant difference in OSI was also observed for the *Taq*I CC genotype; however, the significance disappeared after adjusting for BMI (*p* = 0.026 and 0.057, respectively) (Table 4). Furthermore, a significant difference in the OSI was observed between the calcium-intake groups of the *Apa*I GT genotype, even after adjusting for BMI (*p* = 0.026 and 0.038, respectively) (Table 5). OSI values did not change between the calcium-intake groups in all genotypes of *Fok*I (data not shown).

**Table 3:** Effect of *Bsm*I genotype on OSI between calcium-intake groups

*Bsm*I genotype	Calcium intake	n	Not adjusted		Adjusted^a^	
			OSI	*p*	OSI	*p*
GG	Low	192	2.72 ± 0.02	0.772	2.72 ± 0.02	0.729
	High	207	2.73 ± 0.02		2.73 ± 0.02	
GA	Low	50	2.66 ± 0.03	0.098	2.66 ± 0.03	0.194
	High	42	2.74 ± 0.04		2.73 ± 0.04	
AA	Low	5	2.33 ± 0.06	0.011^b^	2.33 ± 0.06	0.020^b^
	High	3	2.65 ± 0.07		2.65 ± 0.08	

Values are means ± SE. The *p* values indicate the significance of difference in OSI between calcium-intake groups. Differences were assessed using ANCOVA

^a^Adjusted for BMI

^b^*p* < 0.05

**Table 4:** Effect of *Taq*I genotype on OSI between calcium-intake groups

*Taq*I genotype	Calcium intake	n	Not adjusted		Adjusted^a^	
			OSI	*p*	OSI	*p*
TT	Low	184	2.72 ± 0.02	0.586	2.72 ± 0.02	0.613
	High	205	2.73 ± 0.02		2.73 ± 0.02	
TC	Low	60	2.67 ± 0.03	0.221	2.67 ± 0.03	0.333
	High	42	2.73 ± 0.04		2.72 ± 0.04	
CC	Low	3	2.33 ± 0.08	0.026^b^	2.35 ± 0.09	0.057
	High	5	2.63 ± 0.06		2.62 ± 0.07	

Values are means ± SE. The *p* values indicate the significance of difference in OSI between calcium-intake groups. Differences were assessed using ANCOVA

^a^Adjusted for BMI

^b^*p* < 0.05

**Table 5:** Effect of *Apa*I genotype on OSI between calcium-intake groups

*Apa*I genotype	Calcium intake	n	Not adjusted		Adjusted^a^	
			OSI	*p*	OSI	*p*
GG	Low	105	2.72 ± 0.03	0.768	2.72 ± 0.02	0.743
	High	121	2.71 ± 0.02		2.71 ± 0.02	
GT	Low	110	2.69 ± 0.03	0.026^b^	2.69 ± 0.02	0.038^b^
	High	112	2.77 ± 0.03		2.76 ± 0.02	
TT	Low	32	2.69 ± 0.04	0.571	2.69 ± 0.04	0.509
	High	19	2.65 ± 0.06		2.65 ± 0.05	

Values are means ± SE. The *p* values indicate the significance of difference in OSI between calcium-intake groups. Differences were assessed using ANCOVA

^a^Adjusted for BMI

^b^*p* < 0.05

## Discussion

The present study evaluated the VDR genotypes, bone mass, and calcium intake in young Japanese women. As we have previously elucidated the relationship between bone mass and *Cdx2* (rs11568820), a representative SNP of VDR genes [32], the present study focused on four representative VDR gene polymorphisms other than *Cdx2*, including *Bsm*I (rs1544410), *Taq*I (rs731236), *Apa*I (rs7975232), and *Fok*I (rs2228570).

Several prior studies conducted worldwide have reported the relationship between the VDR genotypes and bone mass. Among these, a meta-analysis reported that the bone mass of the spine is significantly lower for the *Bsm*I AA genotype than the *Bsm*I GG and GA genotypes in postmenopausal women (51–75 years) [33]. However, effects of the *Bsm*I AA and *Taq*I CC genotypes on bone mass in Japanese women have rarely been examined because these genotypes are present at a relatively lower proportion than that seen in European countries; e.g., the respective allelic frequencies for the *Bsm*I genotype G and A and *Taq*I genotype T and C in the European population are about 60% and 40%, respectively, compared to about 90% and 10% in the Japanese population [29, 34], indicating a clear racial difference. Furthermore, previous studies involving less than 200 Japanese women showed that only a few of them expressed the *Bsm*I AA and *Taq*I CC genotypes (n = 0–2); hence, the relevant data could not be included in the analyses [20, 21, 35–37]. However, in the present study involving 499 participants, these genotypes were recognized (n = 8 for both), and thus, were included in the analysis. Consistent with the findings obtained in previous studies, the *Bsm*I genotype showed a significant difference in the BMD in young Japanese women; the OSI of the *Bsm*I AA genotype was lower than that for other genotypes.

In addition, the OSI values of the *Taq*I CC genotype differed significantly from those of other genotypes only after adjusting for BMI (*p* = 0.019). Furthermore, previous studies on the *Taq*I genotype and bone mass in premenopausal Japanese women did not report consistent results, with some reporting no significant difference [19, 38–41] while others reporting that participants with the *Taq*I TT genotype display higher bone mass than those with the TC genotype [21, 35]. These studies, however, did not include participants with the *Taq*I CC genotype as their number was limited. In a previous large-scale cohort study (n = 778) on representative samples obtained from Japanese participants, the presence of the *Taq*I genotype, including the CC genotype (n = 11, 1.4%), did not significantly influence BMD in premenopausal women; however, this study was not adjusted for BMI [41]. Therefore, further studies with a sufficient number of participants would be needed to evaluate the effect of the *Taq*I CC genotype on BMD in Japanese women.

The OSI values of the *Apa*I and *Fok*I genotypes were not significantly different in Japanese women in the present study; this observation was consistent with the findings of previous studies on these specific genotypes in Japanese women, which did not report significant differences for genotype and bone mass [19, 35, 39, 41].

We previously reported that the relationship between dietary calcium intake and bone mass differs depending on the VDR *Cdx2* genotype [32]. Furthermore, certain studies have implied that VDR genotypes modulate the calcium absorption rate [42–44]. Gennari et al. [43] reported that postmenopausal participants with the *Bsm*I AA and *Taq*I CC genotypes display reduced intestinal calcium absorption. Consequently, our study showed a significant difference in the OSI values between the calcium-intake groups of the *Bsm*I AA genotype, even after adjusting for BMI (*p* = 0.020). Therefore, this genotype may interact with calcium intake and affect bone mass in young Japanese women. In contrast, no significant effect of the interaction between the *Taq*I CC genotype and the calcium intake on bone mass was observed. Intriguingly, there was a significant difference between the calcium-intake groups of the *Apa*I GT genotype; OSI was higher in the high-calcium intake group. Similarly, a previous study also showed that bone mass in individuals with the *Apa*I GT genotype is elevated concomitantly with high calcium intake; this estimation is made based on the frequency of consumption of seven food groups considered to be rich in calcium: milk, milk products, bean and bean products, meat, fish, dried fish with bone, and green vegetables [39].

The QUS used to measure bone mass in the present study is a non-invasive, painless method that does not involve exposure to radiation [22], and thus it can be applied not only in osteoporosis screening but also in the screening of young Japanese women from the perspective of osteoporosis prevention. According to our results, young Japanese women with low bone mass screened by QUS may be instructed to consume more calcium depending on their VDR gene polymorphism to prevent osteoporosis. Or in near future, if it becomes possible to comprehensively investigate SNPs at a relatively early stage such as at birth and during growth, it will be important to detect the *Bsm*I AA and *Taq*I CC genotypes and attempt to increase bone mass.

Nonetheless, the present study has a few limitations. The phenotypic ratio of VDR gene polymorphisms varies among genotypes; hence, the statistical significance of OSI can differ. Therefore, uniformity in the number of participants is essential. In particular, there were only a limited number of participants for the *Bsm*I AA and *Taq*I CC genotypes and there may have been low variability in these homozygous minor genotypes. Additionally, sample size calculation was not performed in this study because of the limited number of students we were able to recruit. However, to our knowledge, there has been no study that reports the relation between BMD and calcium intake in these genotypes in Japanese women, which may be due to the low proportion of these genotypes in the Japanese population. Therefore, the data from the present study would prove to be valuable. Second, serum vitamin D levels related to bone formation were not considered. Nevertheless, there is reportedly no significant difference in serum vitamin D levels among genotypes for the four VDR gene polymorphisms (*Bsm*I, *Taq*I, *Apa*I, and *Fok*I) [45, 46]. Third, there are several environmental factors that may affect bone mass such as smoking and alcohol consumption, and these factors were not considered herein. Furthermore, a previous study showed that smoking and alcohol consumption does not affect BMD at younger ages [9].

## Conclusions

The present study shows that the VDR *Bsm*I and *Taq*I genotypes are potentially associated with bone mass in young Japanese women. Among them, the *Bsm*I AA genotype may interact with the calcium intake and be related to a lower bone mass under conditions of low calcium intake. In addition, the VDR *ApaI* GT genotype is associated with increased bone mass concomitant with higher calcium intake. Finally, our results may be helpful in the development of a preventive strategy to tackle bone loss and future osteoporosis in young women.

## Data Availability

The datasets used and/or analysed during the current study available from the corresponding author on reasonable request.

## Abbreviations

ANCOVA: Analysis of covariance
ANOVA: Analysis of variance
BMD: Bone mineral density
BMI: Body mass index
FFQg: Food frequency questionnaire based on food groups
OSI: Osteo sono-assessment index
SNP: Single-nucleotide polymorphism
VDR: Vitamin D receptor
